# Dependence of Renal Uptake on Kidney Function in [^68^Ga]Ga-PSMA-11 PET/CT Imaging

**DOI:** 10.3390/diagnostics14070696

**Published:** 2024-03-26

**Authors:** Falk Gühne, Till Schilder, Philipp Seifert, Christian Kühnel, Martin Freesmeyer

**Affiliations:** Clinic of Nuclear Medicine, Jena University Hospital, Am Klinikum 1, 07747 Jena, Germany

**Keywords:** PSMA, eGFR, kidney function, CKD, [^68^Ga]Ga-PSMA-11 PET/CT, isocontour

## Abstract

(1) Background: PSMA ligand PET/CT is increasingly important for diagnostics of prostate cancer and other tumor diseases. In particular, the radiopharmaceutical [^68^Ga]Ga-PSMA-11 is widely used. Besides its tumor-specific binding, the uptake within the kidneys is dominant and seems to visualize the renal cortex specifically. Kidney diseases may alter the uptake of radiopharmaceuticals. Therefore, the correlation between renal uptake in PET/CT imaging and renal function should be investigated. (2) Methods: A group of 103 male patients were retrospectively evaluated for eGFR according to the CKD-EPI equation, tracer uptake intensity (SUV_max_, SUV_peak_, SUV_mean_), the molecular volume of the renal cortex, morphological kidney size, and total renal uptake. Manual and three different computer-assisted contouring methods (thresholds at 50% of SUV_max_, 30% of SUV_max_, and absolute SUV of 20) were used for measurements. Correlations between parameters were calculated using linear regression models. (3) Results: Renal SUV_max_, SUV_peak_, and SUV_mean_ do not correlate with eGFR for manual or computer-assisted measurements. In contrast, molecular cortex volume shows a moderate correlation with eGFR (R^2^ = 0.231, *p* < 0.001), superior to morphological kidney size. A contouring threshold of 30% of SUV_max_ outperformed the other settings for renal cortex volume and total renal uptake. (4) Conclusions: Renal uptake of [^68^Ga]Ga-PSMA-11 cannot predict eGFR, but the functional renal cortex can be quantified by PET/CT imaging.

## 1. Introduction

**Figure 1:**
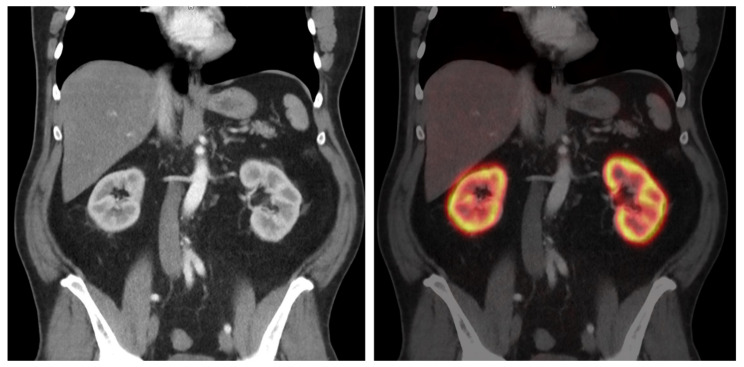
Example of renal PSMA ligand uptake in [^68^Ga]Ga-PSMA-11 PET/CT. (**Left**): Contrast-enhanced CT scan with visual differentiation between renal cortex and medulla in arterial phase. (**Right**): [^68^Ga]Ga-PSMA-11 PET/CT fusion with specific uptake of the radiotracer within the renal cortex. Low uptake in other abdominal organs.

Prostate-specific membrane antigen (PSMA) ligand PET/CT is a relevant and specific multimodal imaging modality for prostate cancer. Its scientific and clinical impact on tumor staging, tumor recurrence, and preparation of radioligand therapy has increased in recent years [1,2,3]. Radioactively labeled ligands bind to PSMA, a protein also known as glutamate carboxypeptidase II, predominantly expressed by prostate cancer cells. In addition, radiopharmaceuticals are also distributed within various organs and compartments, as well as a few other tumor entities [4,5]. Contrary to its name, PSMA is also expressed in other tissues, although usually at lower levels. Since the first use of PSMA ligands in PET/CT imaging, their biodistribution has been evaluated [6,7,8,9]. The organs with the highest uptake are the kidneys, which show a specifically appearing enhancement within the renal cortex (Figure 1). In general, radiotracer accumulation in the kidney is sometimes advantageous, e.g., in renal function scintigraphy, but often disadvantageous in nuclear medicine, e.g., in tumor diagnostics or radioligand therapies. Also, renal failure, especially chronic kidney disease (CKD), is common, in particular, in elderly patients, who are also more often affected by tumor diseases such as prostate cancer.

The apparent visual similarity between ^68^Ga-labeled PSMA ligand PET/CT and [^99m^Tc]Tc-DMSA scintigraphy suggests a possible relationship between renal uptake and renal function. Renal tubular [^99m^Tc]Tc-DMSA uptake is used for regional and relative estimation of renal function and has been shown to correlate with glomerular filtration rate (GFR) [10,11,12]. Only a few nuclear medicine modalities are able to provide a direct estimation of the GFR, while the majority of the techniques are primarily indicative of tubular renal function [13,14]. The comparison between PSMA ligand PET/CT and DMSA scintigraphy has been performed in previous studies, demonstrating the potential of PET/CT to reveal localized functional defects and the relative renal function of both kidneys [15,16]. For this reason, imaging with [^68^Ga]Ga-PSMA ligands could be a valuable addition to conventional radionuclide scintigraphy. On the other hand, PET tracers such as somatostatin receptor analogs have been suggested as potential signs of renal dysfunction [17,18]. Furthermore, comparisons between PSMA ligand uptake of different radiopharmaceuticals and estimated GFR (eGFR) have been performed, with divergent results [19,20,21]

Even though PET/CT imaging is primarily used to detect tumors and inflammation, tissue characterization and information about its dependence on physiological processes can be advantageous for gaining insight into other diseases. [^68^Ga]Ga-PSMA-11 is a widely used radiopharmaceutical for PSMA ligand PET/CT imaging and was recently approved by the U.S. Food and Drug Administration and the European Medicines Agency [22]. The aim of this study was to determine whether a relationship between quantitative renal [^68^Ga]Ga-PSMA-11 uptake and laboratory kidney function existed and if renal dysfunction might be visible in PET scans. Furthermore, multiple PET parameters were compared regarding their correlation to the GFR, and quantification of functional kidney parenchyma was intended.

## 2. Materials and Methods

### 2.1. Inclusion of Patients

All patients who received a PSMA ligand PET/CT for prostate cancer in our tertiary care PET/CT facility within one calendar year were screened for this retrospective observational study. Patients were excluded if laboratory assessment of creatinine was older than 30 days or missing (12%) and if a PSMA ligand other than [^68^Ga]Ga-PSMA-11 had been used (21%). If a patient received more than one PET/CT examination, only the first was evaluated. All examinations were performed on the basis of clinical indications and without study-specific purposes. The justification for the examination in accordance with the German Radiation Protection Ordinance was provided, with all patients giving their written consent to the examination.

### 2.2. Estimation of Kidney Function

Creatinine levels were measured by internal or external laboratories to estimate the glomerular filtration rate. Calculations were performed using the CKD-EPI formula, which was the clinical standard in our institution and which outperformed the accuracy of prior estimation formulas like Modification of Diet in Renal Disease (MDRD) or Cockgroft–Gault, especially in patients with normal to mild-impaired kidney functions [23,24]. For this purpose, the sex, age, and skin color of the patients were considered.

### 2.3. PET/CT Acquisition

The radiopharmaceutical [^68^Ga]Ga-PSMA-11 was manufactured in a Good Manufacturing Practice-qualified radiopharmacy as an in-house production. A patient dose of approximately 250 MBq was administered intravenously, as there were no standard weight-adjusted activities. Additionally, each patient received an intravenous premedication with 20 mg of furosemide. Patients were instructed to drink plenty of water (1 L) prior to the examination; the exact amount drunk was not recorded. The PET/CT scan was accomplished at least 60 min after administration of [^68^Ga]-Ga-PSMA-11. All examinations took place at the same scanner, a Biograph mCT 40 (Siemens Healthineers, Forchheim, Germany), with the same parameters (at least from vertex to mid-thigh, iterative reconstruction, scanning time of 2 min per bed position of 21.6 cm each). For attenuation correction, a low-dose CT was performed using the dose-optimizing application Care Dose 4D (2009 model SOMATOM Definition AS, Siemens Healthineers). The clinical evaluation of the examination and reporting of the results was carried out separately.

### 2.4. Molecular Imaging Parameters Collected

The collection of study-specific parameters was carried out using the dedicated imaging software Syngo.via (Version VB 40, Siemens Healthineers). To avoid inter-observer variability, one examiner performed all assessments. Image analysis was performed without knowledge of the patient’s renal function. Measurements took place either manually or via computer-assisted contouring methods. The standard uptake value (SUV), as a typical measurement on PET/CT imaging for quantifying radiopharmaceutical uptake, was calculated, and body weight was adjusted. The maximum value (SUV_max_) was determined by a spherical volume of interest (VOI) at the place of visually highest uptake. The average value (SUV_mean_) of the renal cortex was manually determined by three identical VOI of 0.5 cm diameter at the upper kidney pole, at the lateral aspect in the middle of the kidney, and at the lower kidney pole. The arithmetical mean of the three SUV_mean_ was calculated.

**Figure 2:**
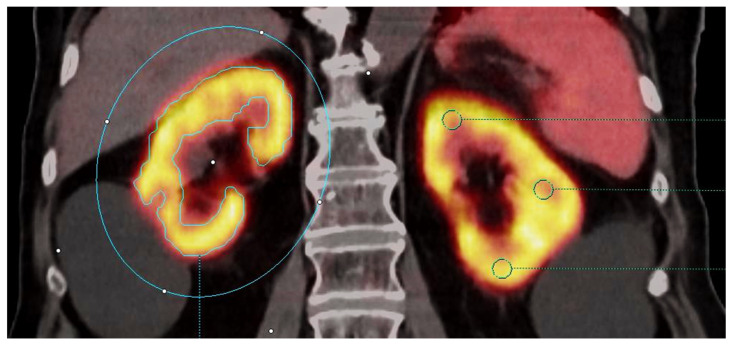
Measurement technique of renal [^68^Ga]Ga-PSMA-11 uptake in PET/CT presented in a frontal plane image. Right kidney (at the left side of the figure): Example of computer-assisted measurement showing a whole kidney VOI with isocontouring of the metabolic volume by a defined threshold (here, IC 30%) to obtain SUV and volume parameters (blue color). Left kidney: Example of manual measurements showing the three 0.5 cm diameter VOI to obtain SUV_mean_ at the predefined locations (green color). Bilateral renal cysts without PSMA ligand uptake were excluded from the measurement.

For computer-assisted contouring methods, a VOI was drawn, taking into account the fact that the entire kidney was covered. Three different thresholds were used to generate a measuring volume within a specific isocontour (IC), including all voxels above 50% of the SUV_max_ (IC 50%), above 30% of the SUV_max_ (IC 30%), and above the absolute SUV of 20 (IC 20 abs.). Thresholds were chosen to aid in the inclusion of the cortex while excluding urinary retention within the renal pelvis. SUV_mean_ and the maximum average SUV in a spherical volume of 1 mL (SUV_peak_) within these contoured volumes were collected. The organ volumes determined by the three respective isocontours were obtained. Furthermore, the products of volumes and SUV_mean_, were calculated, representing the total renal cortex uptake (TRCU), in an analogy to the total lesion glycolysis (TLG) in [^18^F]FDG-PET/CT. To produce an aggregate value for comparison to eGFR, the SUVs of both kidneys were averaged, and the volume and TRCU of both kidneys were added (Figure 2).

### 2.5. Morphologic Parameters Collected

Using the low-dose CT scans, the morphologic size of the kidneys was determined. To calculate the volume, three orthogonal diameters (length, width, height) were measured and inserted into the ellipsoid formula, assuming an ellipsoid shape of the kidney. In addition, the thickness of the renal parenchyma (excluding the renal pelvis) was measured at the lateral aspect in the frontal plane, choosing one representative location. Volume and parenchymal thickness of both kidneys were added for comparison with eGFR. CT and PET scans were evaluated by the same investigator.

### 2.6. Statistics

Statistical analysis was performed using the programming language R (Version 2021, R Core Team, Vienna, Austria). Linear regression was used to compare the variables. The coefficient of determination (R^2^) and the corresponding *p*-value were calculated. To visualize the direction of the correlation, a line was inserted in the graphics using a Pearson correlation. The level of significance was set at *p* < 0.05.

## 3. Results

### 3.1. Patient Characteristics

A total of 103 male patients diagnosed with prostate cancer were included in the study analysis (Table 1). Their PET/CT examinations were performed for different indications: 9 patients on the occasion of initial tumor staging; 76 patients because of biochemical tumor recurrence; and 18 patients for evaluation before planned PSMA radioligand therapy. Accordingly, the numerically assessed tumor burden of the patients was present in varying degrees: 57 patients showed no distinct structural tumor burden; 35 patients showed low tumor burden (one–five manifestations); and only 11 patients showed high tumor burden (>five manifestations). Uptake within the kidneys (manually measured SUV_mean_) showed low dependency from tumor burden (R^2^ = 0.041, *p* = 0.04) with a tendency to lower uptake in patients with high tumor volume.

Renal function measured by laboratory chemistry revealed an estimated glomerular function rate (eGFR) of 76.3 mL/min/1.73 m^2^ on average (range 7.9–105 mL/min/1.73 m^2^). A total of 25% of the patients showed normal kidney function, 57% stage 2 CKD, and 17% stage 3 CKD or higher. Laboratory values were determined at a mean of 11.2 days (range 0–30 days) before PET/CT examinations. After administration of an average of 251.6 MBq [^68^Ga]Ga-PSMA-11 per patient (range 221–276 MBq), the time interval from tracer injection to start of the PET scan was 78.7 min (range 59–133 min).

### 3.2. Quantitative Comparison

**Figure 3:**
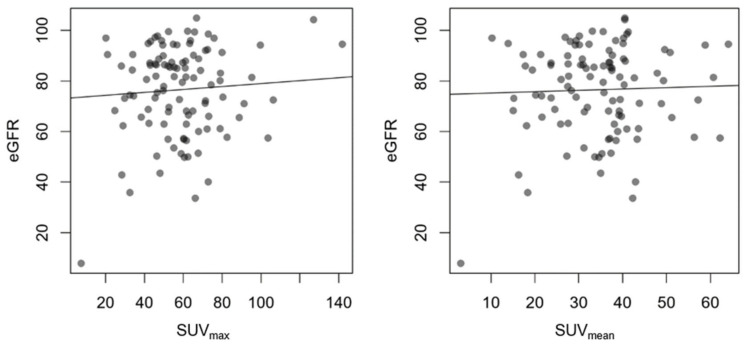
Comparison between eGFR in mL/min/1.73 m^2^ and SUV with linear regression. The circles show the individual measuring points. R^2^ for SUV_max_ 0.004; *p* = 0.515, R^2^ for SUV_mean_ 0.001; *p* = 0.733.

Manually measured uptake was distinctly variable, with an average SUV_max_ of 58.4 ± 21.2 (range 5.9–144.7) and SUV_mean_ of 34.3 ± 12.6 (range 0.5–99.9). Both parameters did not show a correlation with the eGFR (Figure 3).

**Figure 4:**
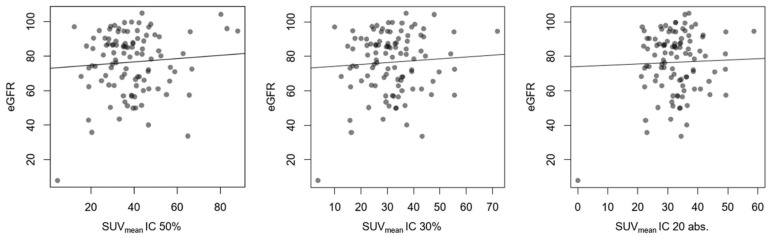
Comparison between eGFR in mL/min/1.73 m^2^ and SUV with linear regression. R^2^ for SUV_mean_ of 50% contouring threshold (IC 50%) was 0.006; *p* = 0.441. R^2^ for SUV_mean_ of 30% contouring threshold (IC 30%) was 0.004; *p* = 0.518. R^2^ for SUV_mean_ contouring threshold SUV 20 (IC 20 abs.) was 0.001; *p* = 0.754.

Regarding the SUV_mean_ measured with the isocontouring method, there was equally no correlation, respectively, for the 50% contouring threshold (IC 50%), the 30% contouring threshold (IC 30%), and the absolute contouring threshold SUV 20 (IC 20 abs.) (Figure 4). The same was observed for the SUV_peak_, IC 50% R^2^ < 0.001, *p* = 0.784, IC 30% R^2^ = 0.002, *p* = 0.668, and IC 20 abs. R^2^ < 0.001, *p* = 0.833, respectively.

**Figure 5:**
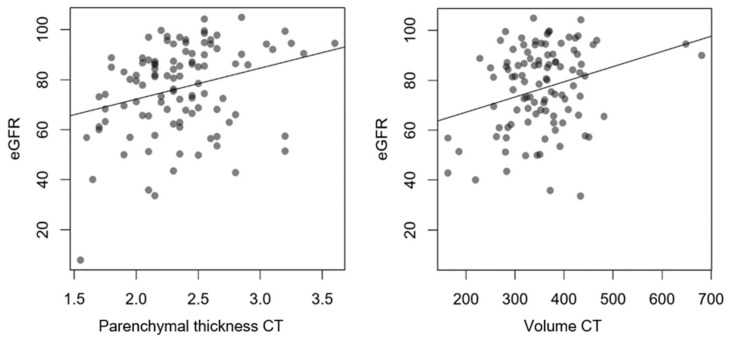
Comparison between eGFR in mL/min/1.73 m^2^ and CT information about kidney size (parenchymal thickness in cm; volume in mL) with linear regression. R^2^ for lateral parenchymal thickness 0.081; *p* = 0.003. R^2^ for kidney volume 0.073 *p* = 0.006.

CT measurements showed an average parenchymal thickness of 2.3 ± 0.4 cm and a mean volume of 353.1 ± 77.5 mL for both kidneys. Six patients only had a single kidney. Morphologically determined kidney size had a low but significant correlation with eGFR, which was present for both lateral parenchymal thickness (R^2^ = 0.081) and total kidney volume (R^2^ = 0.073) (Figure 5).

**Figure 6:**
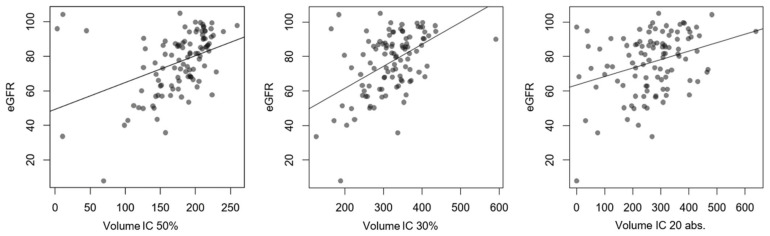
Comparison between eGFR in mL/min/1.73 m^2^ and PET information about volume of renal cortex in mL with linear regression. R^2^ for IC 50% was 0.176; *p* < 0.001. R^2^ for IC 30% was 0.231; *p* < 0.001, and R^2^ for IC 20 abs. was 0.104; *p* < 0.001, respectively.

Molecularly measured kidney volumes had average values of 175.7 ± 46.3 mL for IC 50%, 318.5 ± 69 mL for IC 30%, and 268 ± 114.8 mL for IC 20 abs. A moderate but significant correlation to eGFR could be shown for all administered contouring thresholds, which was higher in comparison to morphological parameters. The best correlation was found for IC 30% (R^2^ = 0.231), followed by IC 50% (R^2^ = 0.176) and IC 20 abs. (R^2^ = 0.104) (Figure 6). In contrast, the total renal cortex uptake (TRCU) for isocontouring thresholds showed lower correlations to eGFR, although they were significant as well, with IC 50% R^2^ = 0.074, *p* = 0.005; IC 30% R^2^ = 0.080, *p* = 0.004; and IC 20 abs. R^2^ = 0.053, *p* = 0.019, respectively. Morphological volume by CT and molecular volume by isocontouring showed the relatively highest correlation when measured using the IC 30% configuration with R^2^ = 0.268, *p* > 0.001. No association between SUV_mean_ and kidney volume was found; e.g., a comparison of manually measured SUV_mean_ and morphologically measured volume resulted in R^2^ = 0.003, *p* = 0.59.

## 4. Discussion

### 4.1. Methods and Limitations

This study restrospectively analyzed clinical data. For this reason, some information was lacking, leading to the exclusion of an important number of patients from the evaluation. Nevertheless, a relatively large number of individuals could be included compared to other authors who investigated smaller cohorts of less than 40 patients [19,20]. There is important heterogeneity in data, especially regarding the period of time between laboratory assessment of eGFR and PET/CT imaging, as well as the uptake time between administration of [^68^Ga]Ga-PSMA-11 and the scanning procedure. Analyses of other PSMA ligands have shown differences in biodistribution depending on uptake time [25]. Other variables, for example, the activity of [^68^Ga]Ga-PSMA-11, might be of negligible impact. The differences in tumor burden might have influenced the correlation because high tumor burden reduced renal uptake in our study and in former studies of other PSMA ligands, such as [^18^F]DCFPyL [26]. Additional parameters, like the individual amount of water consumption in preparation for the examination, were missing and could have possibly had an impact. Regarding the underlying disease, only men have been examined, and the cohort had a relatively high average age of 69 years, whereas the evaluated correlation was only representative of a limited cohort. It is not necessarily possible to transfer the data to the whole population. The eGFR of individuals was distributed relatively symmetrically, also including a relevant number of patients with distinctly decreased kidney function in CKD stage ≥3, which was an advantage of the data shown here.

The creatinine blood-level-based eGFR, regardless of the type of equation, is only an estimation with unknown but existing deviations to real GFR and, therefore, a limitation for comparison to other variables in general. A more exact assessment of GFR, like inulin clearance or creatinine clearance in urine collection, is not accomplished in routine PET/CT imaging and is not available in this evaluation. Advanced eGFR equations, including cystatin C or creatinine and cystatin C in combination, have been shown to be more accurate in estimating GFR but were not used in our data because the creatinine-based CKD-EPI formula was still the clinical standard [27]. Additionally, the eGFR of a specific patient might be variable even in a short period of time and, therefore, different from the values used in this analysis. Reducing the limits of inclusion to a shorter interval (like only 7 or 3 days) in a post-hoc attempt did not influence the correlation substantially, so a significant impact could not be validated. However, time interval remains a significant limitation.

### 4.2. Results and Comparison to the Literature

Neither maximal (SUV_max_, SUV_peak_) nor average (SUV_mean_) quantifications of PSMA ligand uptake showed a correlation to eGFR, regardless of measuring technique. Thus, uptake intensity does not seem to be influenced by kidney function. Measuring methods may affect the correlation in general, especially as SUV_max_ is known to be unreliable in organs with high uptake due to its dependence on single, potentially artificial voxels. For this reason, alternatives like SUV_peak_ or SUV_9_5th have been investigated before to assess high uptake levels [28]. Missing correlation was found for all parameters of uptake intensity measured by SUV in the present study (SUV_max_, SUV_peak_, and several SUV_mean_). Therefore, measuring settings are unlikely to be the main reason for missing coherence to eGFR. In contrast, the molecularly measured volume of the renal cortex is consistent with eGFR, with relevant differences between isocontouring thresholds, favoring IC 30%. However, the dependence is only moderate, indicating a relevant influence of other variables. Although morphologically measured kidney size (volume and parenchymal thickness) also revealed a significant correlation to eGFR, the molecularly measured volume had a higher correlation. It is, therefore, likely to be more representative of functioning tissue than the organ volume, including the renal pelvis and renal medulla. Nevertheless, morphological and molecular volumes are dependent on each other. The TRCU is the most comparable measurement to uptake measured in [^99m^Tc]Tc-DMSA scintigraphy. It showed a significant but very low correlation to eGFR, which was lower than the correlation of volumes. Since molecularly measured volume and SUV_mean_ constitute the TRCU, only the volume seems to be relevant for kidney function in this consideration.

Even though [^68^Ga]Ga-PSMA-11 uptake seems to depict the functional renal cortex specifically, the correlation to kidney function in this analysis is negligible or, at best, moderate. Reasons for this situation might be diverse. On the one hand, the PSMA ligand uptake is probably more representative of tubular function than of glomerular function since PSMA expression was shown to be in the brush-border and apical cytoplasm of proximal tubular cells and not in the glomeruli and Bowman’s capsule [29,30]. Deterioration in glomerular function might not be visual in the quantity of tubular cells, and loss of tubular cells might not be measured by creatinine. Pure tubulopathies are rare and mostly caused by hereditary diseases or nephrotoxic substances, while glomerulopathies are common with inflammatory and non-inflammatory causes [31]. CKD is often multifactorial and is caused by vascular impairment due to arterial hypertension or diabetes mellitus [32,33]. Vasculature itself only shows PSMA expression in tumor neoangiogenesis but not in physiologic kidney vessels [29,34].

[^68^Ga]Ga-PSMA-11 uptake is not only caused by PSMA expression of cells but also by reabsorption and retention of small molecules in tubules and temporarily by urinary excretion of radiotracer within the kidneys. [^68^Ga]Ga-PSMA-11 is a highly hydrophilic molecule and, therefore, is mainly excreted renally [35]. PET/CT imaging at a later point in time could reduce the influence of urinary excretion on renal uptake. This is, however, difficult in practice because of the short physical half-life of ^68^Ga. Other processes of tracer uptake are less time-dependent, so a prolonged uptake time might not have a high impact on the correlation. Concerning all aspects, the intensity of [^68^Ga]Ga-PSMA-11 uptake might be a mixture of renal function and renal dysfunction. This could possibly cancel out the correlation between uptake and function. Other PET radiopharmaceuticals, such as [^68^Ga]Ga-FAPI and [^68^Ga]Ga-DOTATOC, tend to have a negative correlation between renal uptake and eGFR. However, some of the results are controversial [17,19]. The positive correlation between tracer uptake and kidney function is a particular feature of PSMA ligands in PET/CT imaging.

The significance of renal PSMA ligand uptake on kidney function has been evaluated controversially in former studies. Additionally, a multitude of radioactively marked PSMA ligands for PET/CT imaging and laboratory measurements for estimation of GFR exist, which limited comparability. The dominant mode of excretion of radiopharmaceuticals is of particular relevance. In contrast to most PSMA ligands, which are renally excreted, some ligands, e.g., [^18^F]PSMA-1007, are biliary excreted, thereby also affecting biodistribution and tumor detection [36]. Studies regarding [^68^Ga]Ga-PSMA-11 are mostly in agreement with our results. Schierz et al. investigated the correlation by using a different threshold of isocontouring segmentation of an absolute SUV of 10 and a different equation of eGFR by using the Modification of Diet in Renal Disease (MDRD) formula [20]. In accordance with our results, their study revealed no correlation between kidney SUV and eGFR, but they described a significant correlation between kidney-to-bloodpool and kidney-to-liver SUV ratios. To compare these results, we subsequently performed an analysis of both ratios in our data and also found significant correlations. Depending on the SUV measurement settings, R^2^ calculated between 0.11 and 0.15 for the kidney-to-bloodpool ratio and between 0.08 and 0.12 for the kidney-to-liver ratio, all significant at *p* < 0.01. The dependence could, therefore, be reproduced but was weaker than in the publication by Schierz et al. and, generally, to a small extent. Our results differ from theirs, as they did not find any correlation between eGFR and molecular kidney volume or TRCU, while we did. In this regard, it can be suggested that the contouring thresholds used in the current study are more suitable for the segmentation of functional renal parenchyma. Conen et al. found no significant correlation between renal parenchymal uptake of [^68^Ga]Ga-PSMA-11 and GFR. While SUV_max_ displayed no dependence at all, SUV_mean_ showed an insignificant trend toward a positive correlation [19]. However, for [^18^F]PSMA-1007, a strong correlation was investigated in a suitable cohort, showing better results for the correlation with CKD-EPI eGFR than with MDRD eGFR, while the time point of scanning showed no differences in dependency when comparing 1 and 2 h uptake time [21]. The best correlation was found by these authors using the product of SUV_mean_ and segmented volume, similar to the TRCU used in this study, which had less dependence than segmented volume in our data. In contrast, other studies investigated poor correlations between renal [^18^F]PSMA-1007 uptake and eGFR [37]. Moreover, the [^18^F]PSMA-1007 uptake within the bladder also showed no correlation with kidney function [38]. Several efforts have been made to reduce renal uptake, either to improve the tumor-to-kidney ratio and, thus, a sensitivity for renal or kidney-adjacent tumors by evaluating new radiopharmaceuticals [39] or for dosimetric purposes by administering additional drugs in preparation [40]. Renal cancer is an evolving topic in PSMA ligand PET/CT and has been studied in terms of histopathology and imaging [41,42]. Physiological renal uptake should, therefore, be better understood, especially with regard to primary renal tumors.

The information on renal function that can be derived from [^68^Ga]Ga-PSMA-11 PET/CT is limited but is available as an adjunct to oncologic imaging. Dedicated implementation of PET/CT in the diagnosis of renal function or failure seems currently not feasible. But even if the intensity of [^68^Ga]Ga-PSMA-11 uptake does not depict glomerular kidney function, conclusions on local impairments of kidney function or loss of function of a single kidney might be drawn. Also, regarding the assessment of partial kidney function, PSMA ligand PET/CT showed comparable results with standard techniques like [^99m^Tc]Tc-DMSA or [^99m^Tc]Tc-MAG3 scintigraphy [16,37,43].

## 5. Conclusions

It is not possible to predict absolute renal function from retrospective data of routinely performed PET/CT in prostate cancer due to the poor correlation between quantitative [^68^Ga]Ga-PSMA-11 uptake and eGFR. While the intensity of the tracer uptake is mostly independent of the eGFR, the molecularly determined volume of the renal cortex shows a relevant coherence to the renal function. Therefore, tubular renal function can be quantified by [^68^Ga]Ga-PSMA-11 PET/CT. A specific measurement configuration (volume contouring with uptake above 30% of SUV_max_) could be established, which outperformed morphological and other molecular measurements. In our opinion, further studies on kidney diseases and PSMA ligand PET/CT should include detailed clinical information and may better focus on other radiopharmaceuticals and the evaluation of localized functional impairments.

## Figures and Tables

**Table 1:** Patient characteristics (mean values ± standard deviation).

**Participants**	103 males	
**Age**	68.6 ± 7.8 years	
**eGFR**	76.3 ± 17.9 mL/min/1.73 m^2^	>90 mL/min/1.73 m^2^: *n* = 26 60–90 mL/min/1.73 m^2^: *n* = 59 <60 mL/min/1.73 m^2^: *n* = 18
**Time between eGFR** **and PET**	11.2 ± 8.2 days	
**Uptake time of** **[^68^Ga]Ga-PSMA-11**	78.7 ± 16.5 min	

## Data Availability

The raw data can be seen in the graphical representation of the results. In addition, the data can be made available upon reasonable request from the corresponding author.
